# Clay Mineral and Geochemical Proxies for Intense Climate Change in the Permian Gondwana Rock Record from Eastern India

**DOI:** 10.34133/2019/8974075

**Published:** 2019-11-12

**Authors:** Sampa Ghosh, Joydip Mukhopadhyay, Abhijit Chakraborty

**Affiliations:** ^1^Department of Geology, Presidency University, 86/1 College Street, Kolkata - 700073, India; ^2^Department of Geology, Jogamaya Devi College, 92 Shyama Prasad Mukherjee Road, 700026, Kolkata, India

## Abstract

The clay mineral assemblages and geochemical compositions of the Permian Talchir and Barakar mudstones of the Raniganj basin, India, have been used to interpret terrestrial paleoclimate. The Talchir Formation presents unequivocal evidences of the Permian global glacial climate, and the overlying Barakar Formation with braided fluvial deposits immediately follows the glacial amelioration stage to a humid warm climate. Sediments unaffected by burial diagenesis and originated from a similar source under contrasting climates are ideal for developing proxies for substantial climate shift. Illite (28.4-63.8%), illite/smectite (0-58.6%, 40-80% illite), chlorite (0-53.9%), and chlorite/smectite (5.6-29.8%) constitute the clay mineral assemblage in the Talchir Formation whereas illite (5.3-78.2%), illite/smectite (trace-34.1%, mostly 60-90% illite), and kaolinite (36.1-86.8%) dominate the clay mineral assemblage in the Barakar Formation. The Talchir mudrocks are enriched in mobile elements and depleted in alumina w.r.t. PAAS, have relatively higher K_2_O/Al_2_O_3_ ratios (~0.3), high ICV (1.12-1.28), and lower CIA values (52.6-65.1) compared to those of the younger Barakar mudstones. The Barakar mudstones are depleted in mobile elements w.r.t. PAAS, have relatively low ICV (0.33-0.62) and K_2_O/Al_2_O_3_ values (0.11-0.16), and higher CIA values (72.9-88.2). Textural, mineralogical immaturity, and rock fragments of different components of the basement seen in the Talchir sandstones show these sediments being a first-cycle sedimentary deposit. The distinctive clay mineral assemblage and major oxide composition of the Talchir mudrocks attest to a unique low intensity chemical weathering in cold arid climate. Significant presence of kaolinite as well as distinctive geochemical characters of the Barakar mudrocks marks a shift in the paleoclimate from cold arid to humid. This climatic shift is further supported by the proportion and composition of illite/smectite across the formations. The relative proportion of chlorite and kaolinite and composition of illite/smectite therefore closely corroborate the significant climate shift, and such proxies, therefore, are useful indicators of climate extremes in the geological record.

## 1. Introduction

Our Earth preserves a long history of climate change in the stratigraphic record [1]. The earliest known record of such extreme climate dates back to the 2.9 Ga low latitude glacial diamictites from the Mesoarchean Pongola Supergroup in South Africa [2]. In order to understand these changes and to be able to realistically predict future climate change, we need to have a very clear understanding on the tools that can be used for tracking extreme climate change in the past particularly from rock successions. Commonly used proxies include biological ones such as spore pollen, charcoal, foraminifera, coral, and chemical ones such as stable isotopes of oxygen and carbon. Clay mineralogy of sedimentary successions is also believed to provide important clues for conditions of continental weathering and hence climate control [3–6]. But the use of clay minerals as a paleoclimate indicator is somewhat constrained presumably due to limited preservation of ancient sedimentary successions that are minimally affected by postdepositional changes during diagenesis and metamorphism. However, in well-preserved and least-altered rock records, clay mineral assemblages may turn out to be very useful indicators of changing continental weathering regimes and hence the causative climate changes [7, 8]. Although some alteration of clays is prevalent during deposition and early diagenesis [9–11], the clay mineral assemblage may particularly be useful in studying climate proxies from deep-sea drill cores in a higher resolution. Usefulness of such proxies based on clay mineral assemblages may be tested from known stratigraphic records with well-established evidences of abrupt climate change. For example, the Permian-/end-Permian transition witnessed a global climate change from the glaciation stage to the warm humid climate stage in the Gondwana supercontinent (Hambrey and Hartland 1981, [1]. Glacial tillites and supraglacial sedimentary successions in the low latitude Gondwana land are abruptly succeeded by warm climate fluviolacustrine/estuarine siliciclastic deposits [12–15]. Such rock successions of contrasting climatic regimes would be important to formulate suitable lithological proxies for application in climate change studies in the rock record. The extremely different clay mineral assemblages were also documented from the successions of the contrasting Permian climate of the Sydney Gondwana basin, Australia [16]. The major oxide composition of sediments is another important proxy to reveal the paleoenvironmental condition that can be used in conjunction with clay mineral proxies for better resolution in understanding climatic signatures. The bulk chemical changes that take place during weathering are used to quantify the weathering history of sedimentary rocks, primarily to understand past climatic conditions [17, 18]. The use of two lines of evidences moreover would eliminate possible effects of postdepositional changes that may affect interpretation from any one of these proxies when used in isolation.

**Figure 1:**
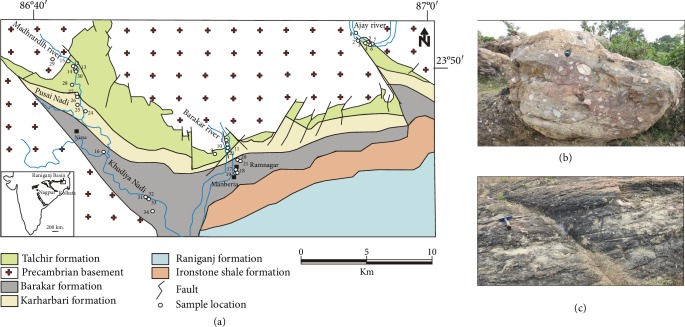
Distribution of the Gondwana rock formations and characteristic field photographs from the Talchir and Barakar Formations in the Raniganj Basin, eastern India. (a) Generalized geological map of the Raniganj basin. Note the distribution of sample locations for the present study. Inset shows the distribution of the major Gondwana basins of Peninsular India. Also note the location of the Raniganj basin. (b) Matrix-supported diamictite of glacial origin from the Talchir Formation. (c) Trough cross-stratified coarse-grained arkosic sandstone from the braided fluvial channel deposits of the Barakar Formation.

Peninsular India is one among a host of best preserved Gondwana successions ([19], Figure 1(a)). The basal Gondwana in India is represented by the Talchir glacial deposits of the early Permian age [15, 20–22]. Ubiquitous glacial depositional (Figure 1(b)) and erosional features in the Talchir rocks unequivocally speak in favour of low latitude glaciation as also recorded in Lower Permian Dwyka glacial deposits in South Africa [23]. The glacial deposits of the Talchir Formation are overlain by a cross-stratified arkose-dominated association (Figure 1(c)) of fluviolacustrine Mid-to-Upper Permian Barakar Formation [15], which is the main repository of coal seams in India as well. The Barakar represents warm humid climate with a drastic change from arid cold climate [24–27]. The Talchir-Barakar transition therefore offers a unique opportunity to study the geological proxies for abrupt climate change that can be used in other successions for unravelling climate change history. In this contribution, we document clay mineral assemblages and geochemical compositions from the mudstones of the Talchir and the overlying Barakar Formations and demonstrate that abrupt climate change from the arid cold glacial period to the warm humid period can be convincingly established particularly in the absence of megascopic evidences.

## 2. Geological Setting, Samples, and Analysis

The samples for the present study come from the Raniganj basin, the easternmost intracratonic rift basin of the Damodar valley basin belt in eastern India (Figure 1(a)). Here the Gondwana Supergroup is about 3,200 m thick and has been classified into six major lithostratigraphic units, namely, Talchir, Barakar, Barren Measures, Raniganj, Panchet, and Mahadeva Formations arranged in ascending order. This basin is about 80 km in length. The Talchir and the Barakar rocks show a regional E-W strike and southerly dip of 5° to 15°. Based on the presence of marine invertebrates (*Praeundolomya subelongata* Dickins, 1963 and *Eurydesma* sp. juv.) and their comparison with faunal assemblages reported from different Gondwana basins of India and Australia, the Talchir Formation of India has been assigned an Early Permian age [21]. On the basis of palynological studies, the top of the Barakar Formation in the Indian Gondwana basins marks the transition from Lower to Upper Permian (Kungurian-Ufimian, [28]).

**Figure 2:**
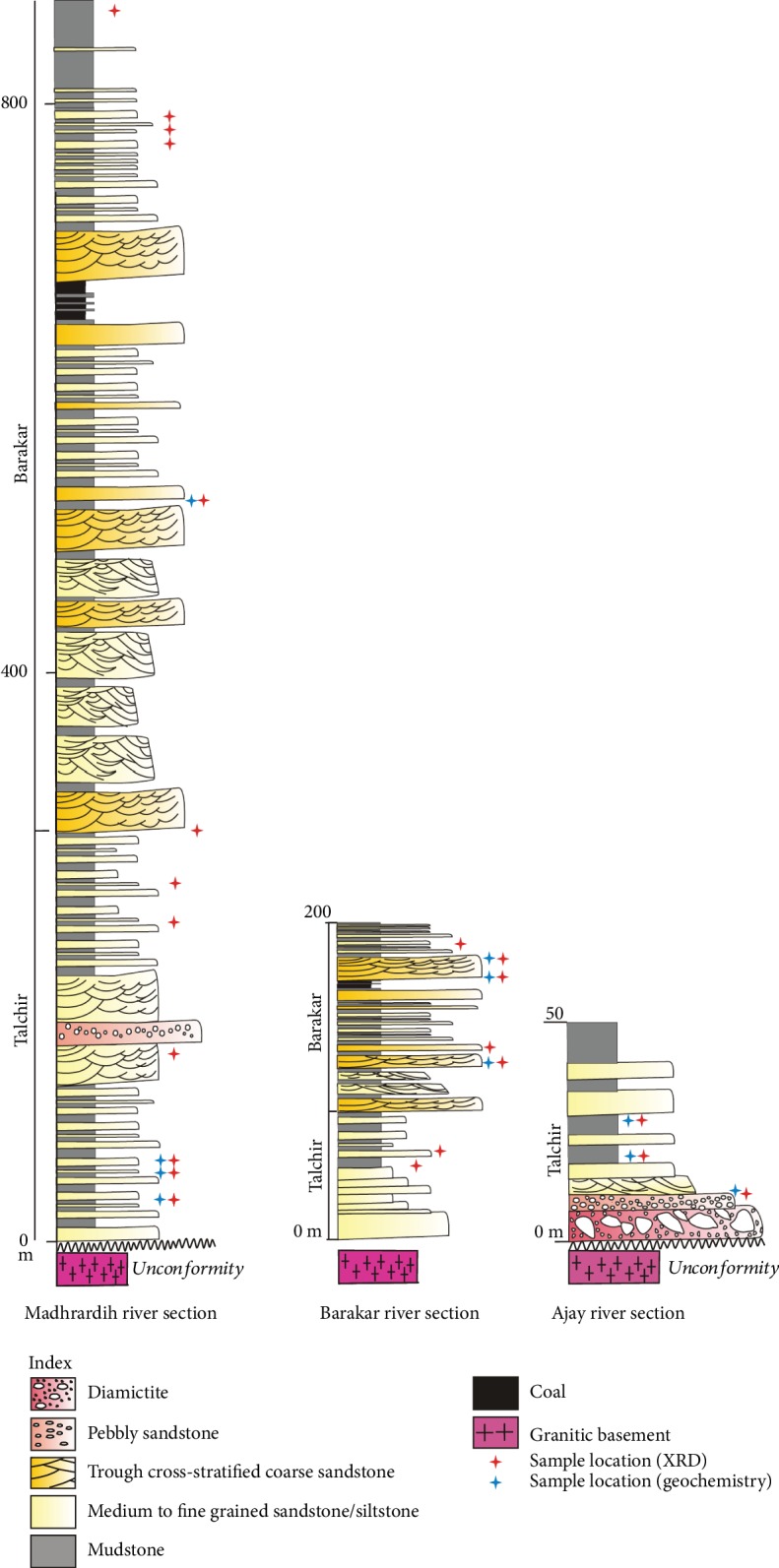
Generalized litholog of the Talchir and Barakar Formations, showing the stratigraphic position of the samples in three different sections.

The lower part of the Talchir Formation is preserved in the Ajay river section (Figure 1(a)). The Talchir Formation here includes glaciogenic diamictites (Figure 1(b)) with faceted pebbles, dropstone boulders, sandstone-mudstone rhythmites ([24, 26, 27], Bhattacharyya and Bhattacharya 2006 2007). The Talchir Formation in the Barakar river section mainly includes sandstone-mudstone heterolithics (Figure 2). The Madhrardih river section includes upper parts of the Talchir Formation, which is dominated by chlorite-rich green sandstone-green mudstone rhythmites grading upwards to the coarse arkosic sandstones of the Barakar Formation. The Barakar Formation in the Madhrardih river section includes a number of fining upward sandstone-shale alternations. The sandstones are thicker in the lower parts. Sandstones in the Barakar Formation are coarse- to medium-grained and feldspathic. The sandstone beds are internally trough cross-stratified and have been interpreted as deposits from braided fluvial environment (Figure 1(c), [25]). The interbedded shales are typical floodplain deposits. Many of the shale intervals host several meters to more than 10 m thick coal seams that constitute the main coal resource of the Raniganj basin [29].

The available paleogeographic reconstructions suggest a very significant latitudinal change of the Indian subcontinent from a position of 60°S latitude in the Lower Permian to 30–40°S latitude by the middle of the Triassic [21]. Therefore, the climatic conditions that influenced the production and deposition of these sediments resulted due to the movement of the Indian subcontinent toward the equator.

The floral, miofloral, and faunal evidences [30–32] as well as the lithologic evidences [33, 34] suggest that the climate changed from glacial cold, semiarid (Talchir) to temperate, humid (Barakar), to warm, semiarid (Raniganj), to warm, semihumid (Panchet), to warm, humid (Mahadeva). It appears that there had been a cyclical change in the amount of precipitation with a gross warming upwards trend [34].

Numerous CO_2_ greenhouse spikes are now recognized during the Permian and Triassic, and these have consequences for stratigraphy and global climate change such as termination of glacial episodes as observed in the Talchir-Barakar transition. In sequences of coal measures, greenhouse crises are marked by paleosols with siderite and berthierine indicative of hypoxia [35]. Increase in CO_2_ results in wetter and warmer local climates [35]. In the present study, the presence of siderite in a carbonaceous mudstone sample of the Barakar Formation ([Supplementary-material supplementary-material-1]) may indicate an increased CO_2_ level.

Samples of sandstones and closely associated mudstones of variable colour were collected from several traverses and mines across the basin and from different stratigraphic levels of the Talchir and Barakar Formations (Figure 1(a)). A total of thirty-nine medium- to coarse-grained sandstone samples were collected from the Talchir and Barakar Formations. Thin sections, perpendicular to bedding planes, were prepared for petrographic studies. The main purpose was to determine the effect of climate and diagenesis on sandstone composition. A total of 24 mudstone samples were subjected to X-ray diffraction (XRD) analysis in order to determine the clay mineral assemblages in each sample ([Supplementary-material supplementary-material-1]). Samples were analyzed from X-ray Minerals, UK, in a Philips diffractometer. Clay minerals are identified and quantified from the diffractograms of the <2 *μ*m clay fraction, and peak intensities are measured and incorporated in a formula to calculate the relative amounts of clay minerals present [36]. Eleven mudstone samples were analyzed by ICP-MS and ICP-ES for determination of trace elements (including REE) and major oxides, respectively ([Supplementary-material supplementary-material-1]). These samples were analyzed from Bureau Veritas Commodities Canada Ltd. Details of the analyses are given in the Appendix. A few selected mudstone samples were observed under scanning electron microscope (SEM) mostly to study the fabric and morphology of mixed layer illite/smectite. The samples were analyzed by Zeiss SEM MA 15 (beam diameter: 20 microns, accelerating voltage: 20 kV) in the laboratory of the Department of Geology, University of Calcutta.

## 3. Results

### 3.1. XRD Analysis of the Mudrocks

**Figure 3:**
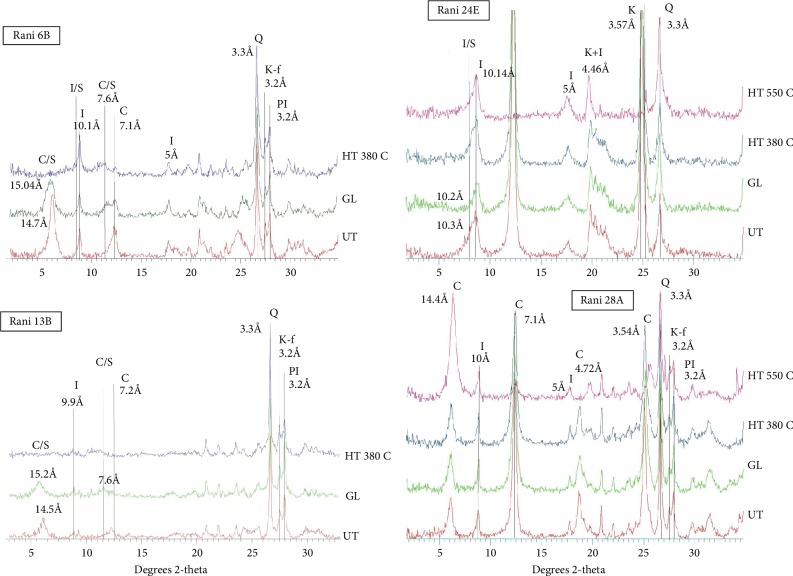
A few representative X-ray diffractograms of clay minerals of the air-dried, glycolated, and heated (380°C) samples (<2 *μ*m fraction) of the analyzed sediments. UT: untreated; GL: glycolated; HT: heated; I: illite; I/S: illite/smectite; C: chlorite; C/S: chlorite/smectite; K: kaolinite; Q: quartz; K-f: K-feldspar; PI: plagioclase. Note that only the most important peaks used in the mineral identification are labeled.

A few representative diffractograms of the air-dried, glycolated, and heated (380°C) samples (<2 *μ*m fraction) of the analyzed sediments are shown in Figure 3. Clay minerals identified mainly include varying proportions of illite, illite/smectite, chlorite/smectite, chlorite, and kaolinite. Siderite (5.3%) is present in a single sample of the Barakar Formation. Plagioclase (2.8-9.3%) and K-feldspar (3.3-15.4%) are present in a number of samples from the Talchir Formation.

**Figure 4:**
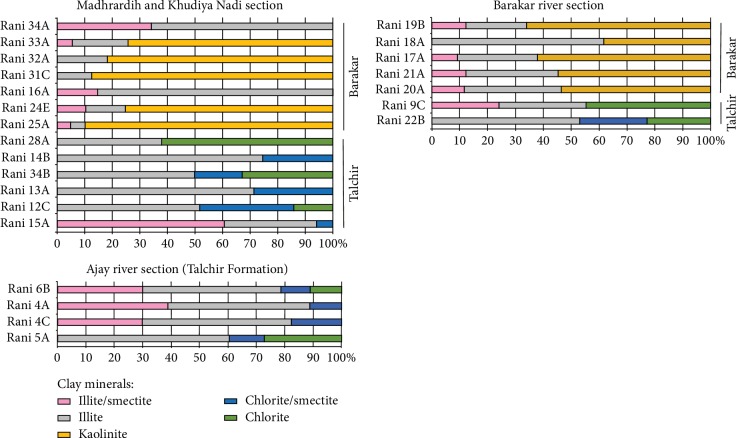
Clay mineral composition of <2 *μ*m fraction of the studied mudstones (graphic representation of [Supplementary-material supplementary-material-1]).

Illite (28.4-63.8%) with some chlorite/smectite (5.6-29.8%, 50-60% chlorite) constitutes the clay mineral assemblage in the Talchir samples. Illite/smectite (I/S with 40-80% illite, R0-R1 ordering, 20.6-58.6%) and chlorite (9.5-53.9%) are present in significant amounts in some of the Talchir samples (Figure 4). In the Barakar mudstones, illite (5.3-78.2%) and kaolinite (36.1-86.8%) are the dominant constituents of the clay mineral assemblage with some illite/smectite (I/S with mostly 60-90% illite, mostly R1-R3 ordering, trace-34.1%, Figure 4).

### 3.2. Geochemistry of the Mudrocks

**Figure 5:**
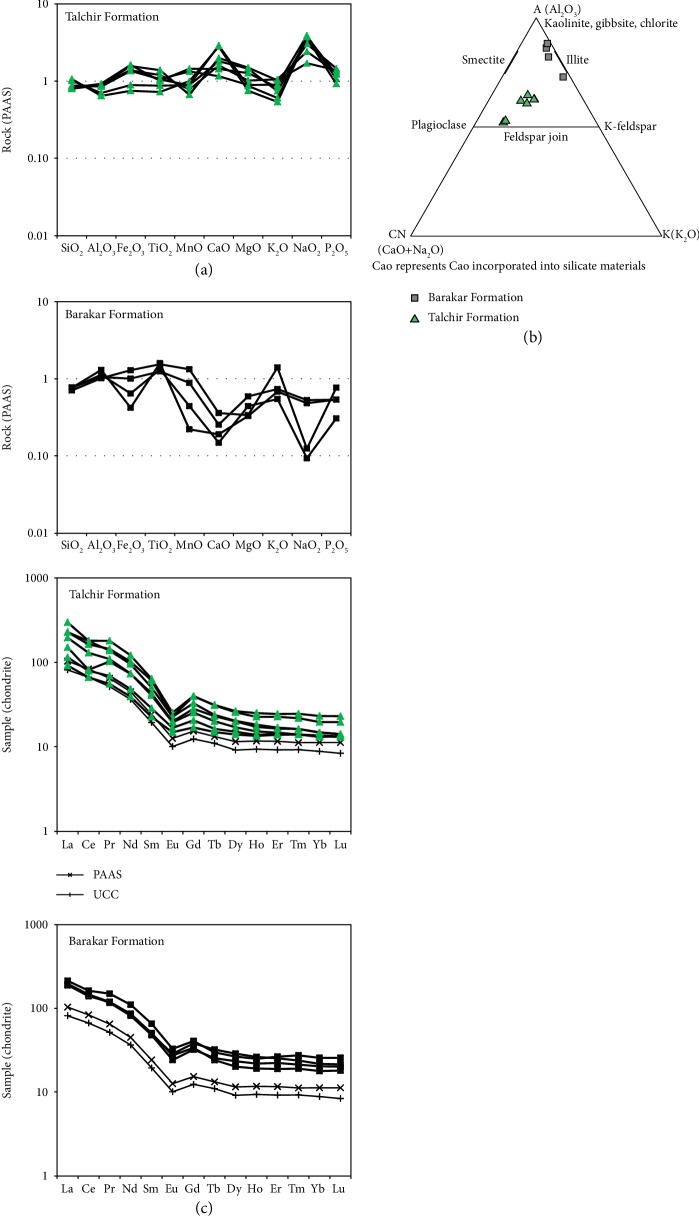
(a) Stratigraphic variation of PAAS-normalized [37] major oxide composition of the mudstone from the Talchir and Barakar Formations. Note the strong enrichment of Na_2_O and depletion of Al_2_O_3_ in the Talchir mudstones and slight enrichment of Al_2_O_3_ coupled with depletion of mobile elements in the Barakar mudstones. (b) Plot in the A-CN-K diagram [18] for the mudstones of the Talchir and Barakar Formations. Note that the Talchir mudstones show a linear trend parallel to the A-CN side of the diagram and the Barakar mudstones plot parallel to the A-K side of the diagram. (c) Chondrite-normalized rare earth element (REE) plots for the Talchir and Barakar mudstones. Note the similarity in REE patterns for both the formations, high LREE/HREE ratio, flat HREE pattern, and pronounced negative Eu anomaly. PAAS and UCC are from Taylor and McLennan [37].

A plot of the major elements, normalized to the Post-Archean Australian Average Shale (PAAS, values after [37], Figure 5(a)), shows slight depletion of Al_2_O_3_ in the Talchir mudstones and slight increase in the Barakar mudstones. Mudstones of the Barakar Formation are generally depleted in most of the major oxides such as SiO_2_, MnO, CaO, MgO, Na_2_O, and P_2_O_5_ relative to PAAS. Enrichment in CaO and Na_2_O coupled with lower values of LOI in the Talchir mudstones suggests the presence of plagioclase. XRD analysis of the Talchir mudstones also confirms the presence of plagioclase.

K_2_O/Al_2_O_3_ values for clay minerals and feldspars range from 0.0 to 0.3 and from 0.3 to 0.9, respectively [38]. The higher values (~0.3, [Supplementary-material supplementary-material-1]) of the Talchir samples are consistent with the presence of K-feldspar and illite as supported by XRD analysis. Low K_2_O/Al_2_O_3_ ratios in the Barakar (0.11–0.16, [Supplementary-material supplementary-material-1]) mudstones are consistent with their kaolinite-rich clay mineralogy. One Barakar sample (Rani 16A) has high K_2_O/Al_2_O_3_ (0.31). XRD analysis of this sample confirms the presence of K-feldspar.

Clay minerals and nonclay silicate minerals are characterized by very different proportions of alumina. On that basis [38], a ratio was defined, the index of compositional variability (ICV): (Fe_2_O_3_ + K_2_O + Na_2_O + CaO + MgO + MnO + TiO_2_)/Al_2_O_3_. It is a measure for the abundance of alumina relative to the other major cations [38] in a rock or mineral. Silica is excluded to eliminate problems of quartz dilution. Nonclay silicates contain a lower proportion of Al_2_O_3_ as compared to that of clay minerals, relative to other constituents, and therefore, have ICV values (0.54–0.87) higher than those of clay minerals (0.03–0.78, [38]). Additionally, there is a compositional gradient within the nonclay silicates: the ICV tends to be highest in minerals high in the weathering sequence of Goldich [39], such as pyroxenes and amphiboles, and decreases in more stable minerals such as the alkali feldspars. The ICV decreases further in the montmorillonite group of clay minerals and is lowest in the kaolinite group of minerals [38]. Because minerals show a relationship between resistance to weathering and ICV, the ICV may be applied to mudrocks as a measure of compositional maturity [38].

The Talchir samples have ICV values in the range of nonclay silicates (1.12-1.28, [Supplementary-material supplementary-material-1]). The Barakar samples show low ICV values (0.33-0.62, [Supplementary-material supplementary-material-1]) mostly in the range of clay minerals except Rani 16A (0.86).

All the mudstone samples are plotted in the A-CN-K (Al_2_O_3_-CaO+Na_2_O-K_2_O) diagram (Figure 5(b)). In the A-CN-K diagram, plots for the Talchir mudstones show a distinct linear weathering trend parallel to the A-CN side, and the pattern is different from those of the mudstones from the overlying Barakar Formation. Mudstones from the younger Barakar Formation show a linear weathering trend along the A-K side in the diagram. In the Permian succession, CIA value is lowest in the Talchir mudstones (52.6–65.1, [Supplementary-material supplementary-material-1]) and increases in the overlying Barakar mudstones (72.9–88.2, [Supplementary-material supplementary-material-1]). Increase in the CIA values possibly reflects increasing proportions of clay minerals with respect to feldspar and also increasing proportion of Al_2_O_3_-rich clay minerals, e.g., kaolinite.

### 3.3. The Trace Element Ratios

Values of the Th/Sc ratio for all of the mudstone samples (0.87–3.25, [Supplementary-material supplementary-material-1]) lie within the expected range for felsic rocks in general (0.84–20.5, [40]) and far exceed the values for mafic source rocks (0.05–0.22). La/Sc and Cr/Th values of the mudstone samples match well with those of sediments derived from felsic source rocks [40]. La/Th values (1.52–2.99, [Supplementary-material supplementary-material-1]) of the mudstones are fairly close to that of UCC (2.7).

All analyzed mudstone samples have total REE abundances similar to higher than that of PAAS. In general, chondrite-normalized REE patterns for the mudstones from both the formations appear similar and are characterized by high LREE/HREE ratio (6.54–11.49, [Supplementary-material supplementary-material-1]), flat HREE pattern and pronounced but variable negative Eu anomaly (Figure 5(c), [Supplementary-material supplementary-material-1]). All the mudstone samples have Eu/Eu^∗^ values lying within a narrow range (0.47–0.76) and fairly close to that of PAAS (0.66).

### 3.4. Petrography of the Sandstones

#### 3.4.1. Talchir Formation

**Figure 6:**
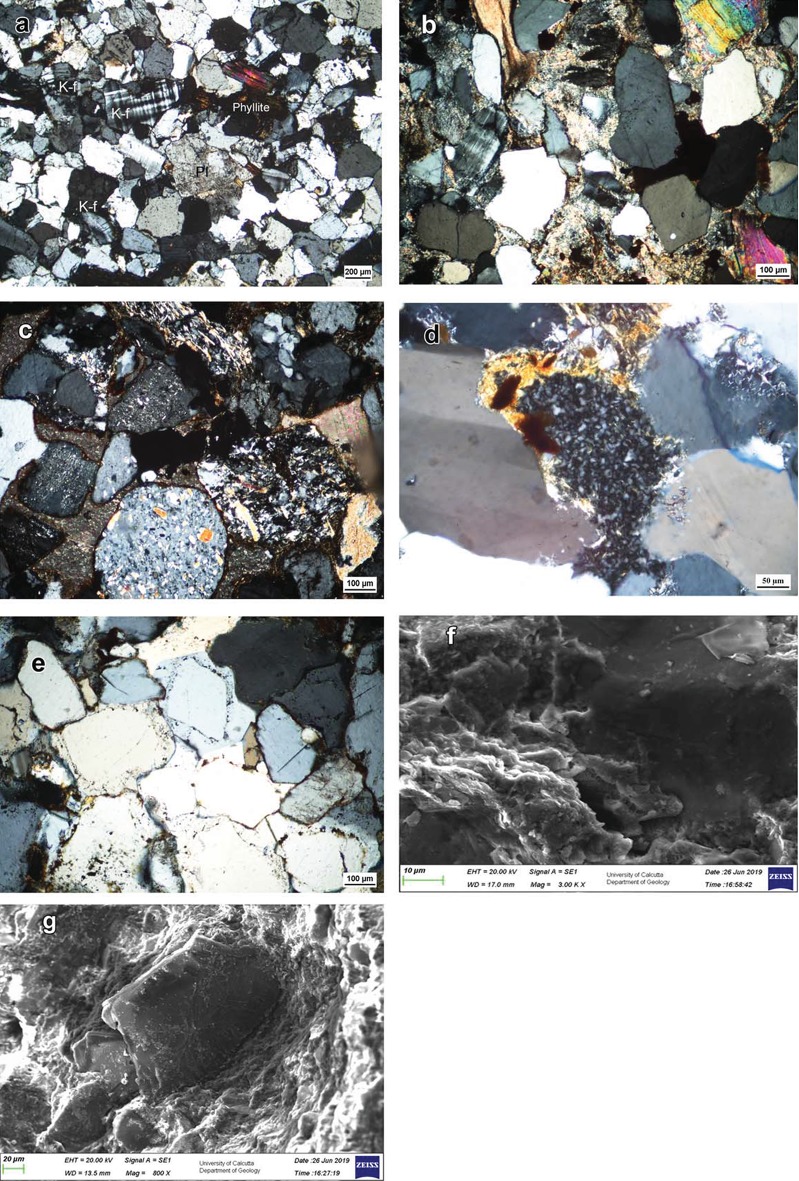
Photomicrographs of sandstones from the Talchir and Barakar Formations. (a) Typical immature Talchir sandstone. Note the common presence of both plagioclase (PI) and K-feldspar (K-f) grains, cross-polarized light. (b) Clayey epimatrix-rich Barakar sandstone with fresh to partly altered feldspar, cross-polarized light. (c) Barakar sandstone showing various stages of K-f alterations, cross-polarized light. (d) Grain-replacive kaolinite cement in the Barakar sandstone, cross-polarized light. (e) Early diagenetic quartz cement in the Talchir sandstone. Note the common occurrence of fusion and interpenetration of overgrowths, cross-polarized light. SEM images for the fabric and morphology of the clay minerals in the mudstones. (f) Illite and mixed layer illite/smectite exhibiting typical bent sheet or detrital plate-like shapes. Note the poor crystallinity of the clays. (g) Warping of the clay particles along the outlines of the adjacent coarser detrital grain.

The Talchir sandstones (pebbly to fine-grained) are texturally and mineralogically immature and moderately to moderately well-sorted. Dominant framework grains include subangular to rounded grains of quartz, plagioclase (both twinned and untwined), K-feldspar (both orthoclase and microcline), biotite, muscovite, and various rock fragments (Q_54-70_F_23-41_R_1-15_, Figure 6(a)). Plagioclase feldspars are generally slightly altered than K-feldspar. Feldspars are relatively fresh barring a few partly altered grains. Rock fragments of granite are the most common ones followed by quartz-mica schist, recrystallized chert, phyllite, intraformational mudclast, etc. Most of the rock fragments are unaltered and easily recognizable. Heavy mineral assemblage includes garnet, epidote, opaque, sphene, and zircon. Clayey protomatrix is present in a small amount (<10%). Carbonate cements are developed locally. Silica cements are also present locally as overgrowth.

Textural and mineralogical immaturity and presence of rock fragments of different components of the Precambrian basement complex attest to the dominance of first-cycle detritus in the Talchir sandstones. The abundance of fresh feldspars (both plagioclase and K-feldspar) and rock fragments and the virtual absence of the epimatrix are in general consistent with the reported cold and arid climate of the Talchir period [30, 31, 33, 34].

#### 3.4.2. Barakar Formation

The Barakar sandstones (coarse- to fine-grained) are texturally immature, moderately to poorly sorted, and matrix-rich (11-25%, Figure 6(b)). Framework grains comprise mostly of angular to subrounded grains of quartz, K-feldspar, muscovite, biotite, and rock fragments of granite, mica schist, biotite-chlorite schist, tremolite-chlorite schist, phyllite, slate, intraformational mudclast, recrystallized chert, etc. (Q_70-95_F_11-28_R_0-5_). K-feldspar (both orthoclase and microcline) is present in different stages of alteration (Figure 6(c)). Biotite is commonly degraded to chlorite. Heavy mineral assemblage includes opaque, sphene, tourmaline, and zircon. These sandstones contain abundant clayey epimatrix with subordinate pseudomatrix. Clay cutans are present locally. Grain- (K-feldspar-) replacive as well as pore-filling kaolinite is well developed (Figure 6(d)). Carbonate cement, silica cement, and iron cement occur locally. These sandstones are mineralogically more mature than those of the underlying Talchir Formation.

The petrographic characters of the sandstones such as the conspicuous absence of plagioclase feldspar, a strong decline in the amount of total feldspar and rock fragments, alteration of K-feldspar and biotite, and common occurrence of epimatrix are consistent with the temperate-humid climatic setting of the Barakar period as suggested by several workers [30, 31, 33, 34].

## 4. Discussion

### 4.1. Source Rock Character and the Role of Diagenesis

The majority of rock fragments in the sandstones are recognizable. A systematic increase in apparent diagenetic destruction of unstable framework grains with depth is not present in the sandstones. In fact, in this case, the most immature sandstone is found at the greatest depth and the most mature one occurs at the youngest stratigraphic level of the basin fill [34]. The Talchir sandstones contain little primary detrital matrix and do not have any authigenic phyllosilicate cements. The clayey epimatrix in the Barakar sandstones is not recrystallized and lacks coarsely crystalline habits. The lack of any preferred orientation of clay minerals in the epimatrix, the presence of the matrix at intergranular contacts, and the clastic texture in the matrix make evident the detrital nature of this matrix. The evidence of early clay infiltration is preserved in the form of occasional cutans, where clay platelets oriented tangentially to the grain surfaces confirm their detrital origin. Another interesting observation is the absence of late-stage diagenetic illite in the studied sandstones. Grain contacts in the studied sandstone samples are simple. Strongly welded and pressure-solved contacts are uncommon.

Simultaneous occurrence of fresh K-f and K-f with various stages of alteration in the Barakar sandstones strongly suggests alteration prior to deposition or during eogenesis. A humid climate, where the groundwater had a low ionic concentration, was favourable for the formation of kaolinite through the replacement of feldspars.

On the basis of mainly textural relations, authigenic quartz and kaolinite cements are interpreted as eogenetic cements. Minor authigenic quartz was possibly formed during early diagenesis before significant compaction as suggested by common fusion and interpenetration of overgrowths (Figure 6(e)). All these above mentioned petrographic indicators strongly rule out any significant involvement of burial diagenesis in the studied succession.

Clay minerals in the mudstones appear poorly crystalline under the SEM. Illite and mixed layer illite/smectite exhibit typical bent sheet or detrital plate-like shapes (Figure 6(f)). Morphologically, it is difficult to distinguish between illite and illite/smectite in our samples as the latter contains a high proportion of illite and therefore essentially exhibit illite-like morphology. We rule out authigenic origin for illite and illite/smectite because of the absence of the characteristic lath shape or filamentous growth. It is generally accepted that the diagenetic illite observed in the SEM would exhibit thread-like or needle-like shapes and would form “bridging” structures between particles [41] as the preservation of these delicate structures precludes extended transport. Moreover, individual flakes of authigenic mixed layer clays commonly show radial arrangement with respect to coarser detrital grain surfaces [42]. Clay particles in our samples, on the other hand, are aligned parallel to the outlines of adjacent coarser detrital grains (Figure 6(g)), which strongly supports their detrital origin. The fabric and the morphology of the clay minerals under SEM certainly confirm absence of significant post depositional alterations and speak in favour of the detrital mode of their origin.

The basement rock types and the paleoflow patterns of the sediments are two main aspects that must be taken into account when determining the influence of source lithology in the resulting clay mineral assemblages of the sediments. Sediment dispersal patterns indicate that the Precambrian terrain straddling the northern margin of the Raniganj basin was the most probable source for the Talchir and the Barakar sediments [27, 43]. The Precambrian basement mostly comprises granites and gneisses (Chotanagpur Granite Gneiss) with subordinate amounts of metamorphic and metasedimentary rocks such as hornblende-schist, quartzite, epidote bearing rocks, actinolite-schist, and mica-schist [20]. The Raniganj basin is small (80 km in length) and the paleocurrent directions for both the formations are very much similar. Paleocurrent directions that were measured from several sections across the basin show broad SW flow direction with minor local deflections for both the formations [27, 43]. It is evident that there was no significant change in the source rocks during the times of sedimentation of these formations. Based on a detailed analysis of detrital quartz types, Suttner and Dutta [34] also concluded that there was little or no change in the nature of the source rocks during the time of Gondwana sedimentation.

The A-CN-K plot (Figure 5(b)) suggests similar sources of granites, granodiorites, gneisses, and metasediments of the basement for both the Talchir and Barakar mudstones and there is no indication of any change in the source rock character between the formations [18].

In general, the mudstones from both the formations have concentrations of high field strength elements (Zr, Nb, Hf, Ta, Th, U, and La; [Supplementary-material supplementary-material-1]) higher than or close to that of UCC, which points towards relatively greater input from felsic source rocks. Enrichment of LIL elements in most of the mudstone samples w.r.t UCC ([Supplementary-material supplementary-material-1]) also suggests a predominantly felsic source. Various trace element ratios (Th/Sc, La/Sc, Cr/Th, and La/Th) for all the mudstones and their REE compositions (Figure 5(c)) strongly support a felsic source. REE compositions of the Raniganj mudstones indicate locally inhomogeneous character. This local inhomogenity is consistent with the short distance of transportation in the Raniganj Gondwana basin.

Since Raniganj basin is a small basin with small catchment area with few rock types; as a consequence, clay mineral assemblages were mostly influenced by significant syndepositional paleoclimatic changes affecting weathering in the source area.

### 4.2. Climate Change as Inferred from the Geochemistry of the Mudstones

Lower proportion of Al_2_O_3_ with respect to PAAS in the Talchir mudstones (Figure 5(a)) and their high ICV values (1.12–1.28) suggest the dominance of nonclay silicates. Preservation of plagioclase and K-feldspar and relatively high K_2_O/Al_2_O_3_ (~0.3), SiO_2_/Al_2_O_3_ (3.05–5.7), and ICV values suggest the compositional immaturity of the Talchir mudstones, lesser intensity of chemical weathering in the source areas, and absence of significant post depositional modifications. In the Barakar mudstones, enrichment of Al_2_O_3_ with respect to PAAS (Figure 5(a)) and lower values of SiO_2_/Al_2_O_3_ (2.02–2.53) indicate dominance of Al-bearing clay minerals, which in turn reflect intense weathering in the source areas. Low K_2_O/Al_2_O_3_ ratios in most of the Barakar (0.11–0.16) mudstones caused by the presence of high proportion of kaolinite indicates moderate to intense chemical weathering of the source rocks [44]. Low ICV values (0.33–0.62) of most of the Barakar mudstones also support compositional maturity and intense chemical weathering in the source areas. One mudstone from the lower part of the Barakar Formation is relatively immature as shown by high ICV (0.86), high K_2_O/Al_2_O_3_ (0.31), and preservation of K-f, which indicates burial diagenesis was negligible.

The weathering pattern as shown by the Talchir mudstones in the A-CN-K plot (Figure 5(b)) and the relatively lower CIA values (52.6–65.1) attest to a low intensity of chemical weathering. Sediments were derived from zones III and IV of a typical weathering profile. Temperate, humid climate during the period of deposition of the Barakar Formation increased chemical weathering, and hence, weathered canopies were developed on the bedrock. This is reflected in the plots of the younger Barakar mudstones in the A-CN-K diagram, where the muds were derived from zones II and III of a weathering profile and their relatively higher CIA values (72.9-88.2). It is obvious that chemical weathering had the major influence in increasing the mineralogical maturity of the sediments from the underlying Talchir Formation. Erosion produced muds more aluminous than those previously produced during the Talchir period. The plot indicates non-steady-state weathering for both formations, where material removal rate exceeds the production of weathering products [18]. This is in agreement with the relatively active tectonic setting of both the formations. The clearly demonstrated change in the intensity of chemical weathering from the Talchir Formation to the Barakar Formation is consistent with climate change from cold, arid to temperate, humid.

### 4.3. Climate Change as Inferred from the Clay Mineral Assemblages of the Finer Fraction of the Mudstones

The clay mineral assemblages in the <2 *μ*m fraction of the Permian mudstones from the Raniganj basin are ideal for the interpretation of the paleoclimate as these rocks originated from similar source rocks under very contrasting climatic conditions and the effects of burial diagenesis is negligible. This is further supported by the significant presence of K-feldspar (max. 15.4% in Rani 4C) and plagioclase (max. 9.3% in Rani 4C) in the <2 *μ*m fraction of the Talchir mudstones.

Illite is fairly abundant in both the Talchir (28.4-63.8%) and the Barakar mudstones (5.3 to 78.2%, [Supplementary-material supplementary-material-1]), which is in conformity with significant amounts of detritus coming from granitic and gneissic sources (Chotanagpur Granite Gneiss, compare [7]). In addition to that, weathering of phyllites and schists possibly contributed some illites. Illites are mostly mechanically ground primary clay minerals and stay preserved after physical weathering processes such as glacier action [4]. The illite is structurally similar to muscovite and is most commonly inherited by sediments through physical weathering and slight chemical modification of the muscovite in the source area. Under the glacial climatic regime during the Talchir time, only mechanical weathering was significant and led to the preservation of signature of the local basement in fine sediments as revealed by illites.

Chlorite is significant in the majority of the samples from the Talchir Formation (9.5-53.9%) and is considered detrital. Chlorite may be inherited directly from the parent igneous rocks subjected to mild hydrothermal activity or from the low-grade pelitic metasediments in the source. As it is unstable under warm, humid, and strongly acidic conditions, the occurrence of detrital chlorite in the Talchir samples implies at least either a cool or arid weathering environment. Chlorite at places transformed to mixed layer chlorite/smectite as a result of surface exposure.

In general, smectites commonly form in soils under seasonally contrasted climate and/or above basaltic bedrock and then transform into I/S R0 with incipient burial [45]. Mafic rocks are virtually absent in the source area and burial diagenesis is negligible. There is a demonstrable inverse relationship between the composition and ordering of the mixed layer I/S clays with depth. The I/S clays in the younger Barakar mudstones are more illitic and ordered (mostly 60-90% illite with R1 to R3 ordering) compared with the older Talchir mudstones (I/S clays with 40-80% illite, R0-R1 ordering), which rules out diagenetic conversion of smectite to illite/smectite with increasing burial depth.

Since we ruled out diagenetic conversion of illite, paleoflow pattern, petrography, and whole-rock geochemistry of the sediments confirmed similar/single source, the mixed layer illite/smectite is most likely produced by degradation of the abundant detrital illite. We consider the illite/smectite a weathering product of the illite, and this weathering is a multistage chemical process, where smectitization occurs through the intermediate I/S mixed layer clay mineral phases [46]. In cold glacial climate during the deposition of the Talchir rocks, rates of weathering tend to be reduced. Weathering in cold areas is quite unique from other climates and commonly results in the formation of interstratified clays [47]. As in the deposits of the Talchir Formation, a significant amount of mixed layer illite/smectite and smectite has been reported by others [48, 49] to be present in glaciogene sediments. The percentage of illite in the I/S clays is significantly different between the Talchir and Barakar Formations. More illitic I/S clays are found together with the kaolinite-rich Barakar sediments. This association is interesting. The presence of kaolinite in the Barakar mudstones reveals a highly weathered sedimentary formation. When climatic conditions became temperate-humid during the Barakar period, the intense chemical weathering of the basement rocks resulted in leaching of most of the cations and produced large amount of kaolinite [49]. Appearance of profuse Gondwanic flora enhanced the acidic character of the soils, which favoured hydrolysis of aluminosilicates, e.g., feldspars, micas, and hornblendes [4], that are very common in the granitic rocks of the Precambrian basement complex. Similar kaolinite dominant assemblage has been reported from the coal and coaly mudstone bearing sediments of the Oligocene-Miocene succession in the As Pontes basin, Spain, and the Carboniferous coal bearing sediments of the Paganzo Group in Argentina [7, 50]. As temperate-humid climate with good drainage conditions during the time of deposition of the Barakar Formation was favourable for leaching, it was not conducive for smectitization. Evidently, the mixed layer I/S clays occur in less proportion (trace-34.1%) and are more illite-rich (60-90% illite) and occur in association with kaolinite. Differential weathering caused by a pronounced change in climate during the deposition is clearly evident in the compositional variation of the mixed layer illite/smectite across the formations.

## 5. Conclusion

The distinct differences in the clay mineral assemblages and major oxide composition of the mudrocks between the Talchir and the Barakar Formations, where diagenesis has not been severe enough to significantly modify the predepositional features, closely correspond to their climatic conditions during deposition as source lithology did not change much across the formations. The basement is mostly represented by granites and gneisses (Chotanagpur Granite Gneiss) with minor metamorphic and metasedimentary rocks. The abundance of illite in both the formations is consistent with their dominant granitic and gneissic sources. The cold arid climate for the Talchir Formation is well corroborated with illite, chlorite/smectite, illite/smectite (40-80% illite), and chlorite dominant assemblage and major oxide compositions (enrichment of mobile elements, depletion of alumina, high ICV (1.12-1.28), and relatively low CIA values (52.6-65.1)) of the Talchir mudstones. The mixed layer illite/smectite is a degradation product of the abundant detrital illite. The chlorite at places transformed to the mixed layer chlorite/smectite as a result of surface exposure. The warm humid condition in the Barakar Formation is mainly represented by kaolinite, illite, and illite/smectite (mostly 60-90% illite) dominant assemblage and is further confirmed by the relatively high CIA values (72.9-88.2), low ICV (0.33-0.62), low K_2_O/Al_2_O_3_, and enrichment of Al_2_O_3_ in the mudstones. The presence of significant kaolinite in the Barakar Formation suggests intense chemical weathering, which is consistent with transition from cold arid to humid, which during the Barakar period favoured leaching and prevented illite to transform into smectite. Hence, I/S clays are less abundant and more illitic (mostly 60-90% illite) in the Barakar mudstones. The paleoclimate analysis from the clay mineral assemblages and the major oxide composition of the mudstones is consistent with the previously published interpretations from several other proxies. The abundance of chlorite or kaolinite and the proportion and composition that essentially indicate the maturity of transformation of mixed layer I/S clay minerals therefore can be a useful indicator of climate extremes in geological record. The clay mineralogy and geochemical proxies therefore may serve as useful tools to decipher climate change.

## Appendix

### A.1. Method

For XRD analysis, the sample is first disaggregated gently using a pestle and mortar. A 2 g split of this material is then “micronised” using a McCrone Micronising Mill to obtain an X-ray diffraction “powder” with a mean particle diameter of between 5 and 10 microns. The sample as slurry is dried and recrushed to a fine powder producing a randomly oriented sample for presentation to the X-ray beam.

Separating the <2 *μ*m fraction is achieved by ultrasound and centrifugation. The samples are analyzed as untreated clay, after saturation with ethylene glycol vapour overnight and following heating at 380°C for 2 hours, with a further heating to 550°C for one hour. The initial scan for these four treatments is between 3° and 35° 2*θ* at a step size of 0.05°/sec using X-ray radiation from a copper anode at 40 kV, 40 mA. The untreated sample is also analyzed between 24 and 27° 2*θ* at a step size of 0.02°/2 sec to further define kaolinite and chlorite peaks. Illite is identified by the presence of 10.1 Å peak. This profile is unaffected by ethylene glycol solvation and heating to 380°C. Kaolinite is identified by the presence of 7.2 Å and 3.58 Å peaks. The peaks of kaolinite remain unaffected upon ethylene glycol treatment but get destroyed on heating above 450°C. The 002 reflection of kaolinite is at 3.57 Å (24.9° 2*θ*) while chlorite has the 004 reflection at 3.53-3.52 Å (25.1°-25.2° 2*θ*). Chlorite has a series of reflection that kaolinite has not such as the 001 reflection at 14.2 Å (6.2° 2*θ*) and the 003 at 4.7 Å (18.8° 2*θ*). Heating at 380°C does not affect chlorite and kaolinite except for a reduction in the intensity of kaolinite. Heating at 550°C collapses kaolinite completely while chlorite peaks after these treatments are much weakened, except for the 001 reflection that usually increases and shifts to 13.8 Å. The most diagnostic reflections (004) for chlorite/smectite are about 11.3° 2*θ* or 7.8 Å (50% chlorite)/11.7° 2*θ* or 7.6 Å (60% chlorite) after ethylene glycol solvation. The presence of chlorite/smectite is confirmed as the diffraction pattern indicates expansion with ethylene glycol solvation (12.5° 2*θ* after glycolation).

The samples that show a low, broad diffraction peak, at about 6° 2*θ* (d spacing around 15 Å), were solvated in ethylene glycol. After the treatment, the low broad diffraction peak at about 6° 2*θ* changed to lower 2*θ* values (about 5.2° 2*θ*) and the d spacing increased to about 17 Å. This peak indicates that a smectite component is surely present. The samples, which produce a peak near 5.2° 2*θ* in ethylene glycol solvation, are rich in smectite. A reflection near 9° 2*θ* and 16 to 17.7° 2*θ* in air-dried samples indicates the presence of illite/smectite. The presence of illite/smectite is confirmed as there is a movement of the peak at 8.8° 2*θ* after glycolation. The 5.2° peak in the glycolated sample becomes broader and weaker as the proportion of illite increases and nearly disappears for samples containing 70-90% illite in the mixed layer illite/smectite. Peaks are sharper for the ordered structure. The ordering type is determined by the position of the reflection between 5 and 8.5° 2*θ* for ethylene glycol solvated preparations. A reflection at 5° 2*θ* indicates random interstratifications, and one near 6.5° 2*θ* indicates R1 ordering. The latter reflection becomes broad and weak and does not move much as the composition becomes more illitic [51]. Upon heating, the diffraction patterns resemble a pure illite (10 Å structure) for the samples containing mixed layer illite/smectite.

Peak intensities are measured and incorporated in a formula to calculate the relative amounts of clay minerals present [36].

For the geochemical analysis of whole rock, the mudstone samples were powdered to −200 mesh using a chromium free tool steel mill (Pulverisette 9 Fritz-GMBH). Major element analyses were carried out by ICP-emission spectrometry (ICP-ES). The samples were analyzed on a SPECTRO ARCOS spectrometer. A completely revised generator with air-cooling, a 5 kW ceramic tube, and a solid-state power supply provide the SPECTRO ARCOS with absolutely stable plasma conditions. A free running oscillator with a resonance frequency of 27.12 MHz and a power output of 0.7 to 1.7 kW is employed. Electrical requirements are 230 VAC ± 5%, 50/60 Hz, and approx. 4.5 kVA power consumption. A classical whole-rock analysis for 11 major oxides and several minor elements by ICP-emission spectrometry was done following a lithium borate fusion and dilute acid digestion. Loss on ignition (LOI) was determined by sintering at 1000°C, and LECO analysis was done for total carbon and sulphur. Certified sulphur reference materials (GS 311-1, GS 910-4) were used as standards for this analysis. Certified control values are given in [Supplementary-material supplementary-material-1]. Accuracy and precision of ICP-ES analysis is reported as better than 1.5% by ACME Labs. Standard SO-18 (mixed rock matrix) was used for ICP-ES analysis.

Analyses of trace element concentrations including rare earth elements (REE) were carried out using a NexION 300 ICP-MS. Analytical conditions for NexION 300 ICP-MS were 22 A maximum continuous current, 200-240 operating voltage, and 50/60 Hz operating frequency. For each sample, 2 to 3 replicate analyses were carried out in order to check that precision and accuracy were within acceptable limits. The precision was <5% RSD with comparable levels of accuracy. The precision and accuracy are based on multiple analyses of international rock standard SO-18. Results of analyses of the standard samples are given in [Supplementary-material supplementary-material-1].

A good measure of the degree of chemical weathering is given by the chemical index of alteration (CIA, [18]) using the formula CIA = [Al_2_O_3_/(Al_2_O_3_ + CaO^∗^ + Na_2_O + K_2_O)] × 100 (in molecular proportions), where CaO^∗^ is the amount of calcium incorporated in the silicate fraction of the rock. The bulk compositions for sediments and rocks are plotted in the same manner as minerals. The weight percentages of bulk chemical analyses are first converted to moles of oxides (only Na_2_O, CaO, K_2_O, and Al_2_O_3_ need conversion for this index). The mole percentages of these oxides are then calculated and plotted on the A-CN-K diagram. Any CaO associated with calcite, dolomite, or apatite should be removed from the total CaO so that the remainder reflects CaO in silicates only [18].

## Supplementary Materials

Supplementary Materials Table S1: X-ray diffraction analysis of the mudstones (<2 *μ*m fraction). Table S2: major oxide (weight %) and trace element including rare earth element concentrations (ppm) of the mudstones. Table S3: ICV, K_2_O/Al_2_O_3_, SiO_2_/Al_2_O_3_, CIA, and trace element ratios in the mudstones from the Raniganj basin compared with the analogous ratios in sediments derived from felsic rocks, mafic rocks, and upper continental crust. Table S4: analyses of rock standards [40, 52].
